# Trans-Lamina Cribrosa Pressure Difference and Open-Angle Glaucoma. The Central India Eye and Medical Study

**DOI:** 10.1371/journal.pone.0082284

**Published:** 2013-12-06

**Authors:** Jost B. Jonas, Vinay Nangia, Ningli Wang, Karishma Bhate, Prabhat Nangia, Purna Nangia, Diya Yang, Xiaobin Xie, Songhomitra Panda-Jonas

**Affiliations:** 1 Suraj Eye Institute, Nagpur, India; 2 Department of Ophthalmology, Medical Faculty Mannheim of the Ruprecht-Karls-University of Heidelberg, Mannheim, Germany; 3 Beijing Tongren Eye Center, Beijing Tongren Hospital, Capital Medical University, Beijing Ophthalmology and Visual Sciences Key Laboratory, Beijing, China; Zhongshan Ophthalmic Center, China

## Abstract

**Purpose:**

To assess associations of the trans-lamina cribrosa pressure difference (TLCPD) with glaucomatous optic neuropathy.

**Methods:**

The population-based Central India Eye and Medical Study included 4711 subjects. Based on a previous study with lumbar cerebrospinal fluid pressure (CSFP) measurements, CSFP was calculated as CSFP[mmHg] = 0.44 Body Mass Index[kg/m2]+0.16 Diastolic Blood Pressure[mmHg]−0.18×Age[Years] −1.91. TLCPD was IOP–CSFP.

**Results:**

Mean TLCPD was 3.64±4.25 mm Hg in the non-glaucomatous population and 9.65±8.17 mmHg in the glaucomatous group. In multivariate analysis, TLCPD was associated with older age (*P*<0.001; standardized coefficient beta:0.53; regression coefficient B:0.18; 95% confidence interval (CI):0.17, 0.18), lower body mass index (*P*<0.001; beta: −0.28; B: −0.36; 95%CI: −0.38, −0.31), lower diastolic blood pressure (*P*<0.001; beta: −0.31; B: −0.12; 95%CI: −0.13, −0.11), higher pulse (*P*<0.001; beta:0.05; B:0.02; 95%CI:0.01,0.2), lower body height (*P* = 0.02; beta: −0.02; B: −0.01; 95%CI: −0.02,0.00), higher educational level (*P*<0.001; beta:0.04; B:0.15; 95%CI:0.09,0.22), higher cholesterol blood concentrations (*P*<0.001; beta:0.04; B:0.01; 95%CI:0.01,0.01), longer axial length (*P* = 0.006; beta:0.03; B:0.14; 95%CI:0.04,0.24), thicker central cornea (*P*<0.001; beta:0.15; B:0.02; 95%CI:0.02,0.02), higher corneal refractive power (*P*<0.001; beta:0.07; B:0.18; 95%CI:0.13,0.23) and presence of glaucomatous optic neuropathy (*P*<0.001; beta:0.11; B:3.43; 95%CI:2.96,3.99). Differences between glaucomatous subjects and non-glaucomatous subjects in CSFP were more pronounced for open-angle glaucoma (OAG) than for angle-closure glaucoma (ACG) (3.0 mmHg versus 1.8 mmHg), while differences between glaucomatous subjects and non-glaucomatous subjects in IOP were higher for ACG than for OAG (8.5 mmHg versus 3.0 mmHg). Presence of OAG was significantly associated with TLCPD (*P*<0.001; OR:1.24; 95%CI:1.19,1.29) but not with IOP (*P* = 0.08; OR:0.96; 95%CI:0.91,1.00). Prevalence of ACG was significantly associated with IOP (*P* = 0.04; OR:1.19; 95%CI:1.01,1.40) but not with TLCPD (*P* = 0.92).

**Conclusions:**

In OAG, but not in ACG, calculated TLCPD versus IOP showed a better association with glaucoma presence and amount of glaucomatous optic neuropathy. It supports the notion of a potential role of low CSFP in the pathogenesis of open-angle glaucoma.

## Introduction

The trans-lamina cribrosa pressure difference (TLCPD) has been defined as the difference between intraocular pressure (IOP) minus cerebrospinal fluid pressure (CSFP), while the IOP, in a strict physical sense, is the transcorneal pressure difference between the intraocular compartment and the surrounding external atmosphere [1], [2]. Since the lamina cribrosa of the optic nerve head, as the presumed site of glaucomatous damage to the optic nerve, is the pressure shed between the intraocular compartment and the retrolaminar compartment with the cerebrospinal fluid space, it has been discussed that the TLCPD as compared with the IOP may be more important for the pathophysiology of the optic nerve head including the development of glaucomatous optic neuropathy [3]–[11]. To test this hypothesis, we examined whether TLCPD as compared with IOP is better correlated with the presence of glaucoma and with indices indicating the amount of glaucomatous optic nerve damage. We differentiated between open-angle glaucoma (OAG) and angle-closure glaucoma (ACG), since in OAG extraocular factors may be involved in the pathogenesis of the optic nerve damage, while in ACG, the reason for the increased IOP and the damage of the optic nerve has been considered to be located intraocularly. We chose a population-based study design to avoid the potential bias due to referral-related selection of study participants. Since measurement of CSFP as one part of the equation to calculate the TLCPD is invasive, we estimated the CSFP based on diastolic blood pressure, age and body mass index, using a formula which was derived in a previous investigation on the relationship between these three parameters [12].

## Methods

The Central India Eye and Medical Study (CIEMS) is a population-based cross-sectional study performed in 8 remote villages close to so called tribal regions in Central India between 2006 and 2008 [13], [14]. Of a total population of 13,606 villagers, 5885 subjects met the inclusion criterion of an age of 30+ years. Of the 5885 eligible subjects, 4711 subjects (2191 men (46.5%)) participated (response rate: 80.1%). The mean age was 49.5±13.4 years (median: 47 years; range: 30–100 years), and the mean reported monthly income was 1584±1233 rupees (1 US dollar equals roughly 50 rupees); the rate of illiteracy was 35%. Among the 1174 non-participants were 685 (58.3%) men; the mean age was 48.6±14.1 years (median: 45 years; range: 30–95 years). The group of study participants and the group of non-participants did not differ significantly in age (P = 0.06), while the proportion of men was significantly (P<0.001) higher in the group of non-participants.

All examinations were carried out during a day-long stay at a hospital to which the study participants were transported by a bus. Trained social workers asked questions about regarding background, living conditions, tobacco use and alcohol consumption, and any known diagnosis of major systemic diseases. Pulse, arterial blood pressure, and body height and weight were recorded. One-and-a-half hours after a standardized lunch, blood and urine samples were obtained and biochemically analyzed. A detailed ophthalmologic examination included refractometry for visual acuity assessment, frequency-doubling perimetry, Goldmann applanation tonometry, slit lamp biomicroscopy, sonographic corneal pachymetry and biometry (Pacscan; Sonomed, Lake Success, NY, U.S.A.), gonioscopy, digital photography of the cornea, lens, optic disc and macula, and confocal laser scanning tomography (HRT, Heidelberg Engineering, Heidelberg, Germany) of the optic disc. Glaucoma was defined by a glaucomatous appearance of the optic disc. The optic nerve head was glaucomatous (1) if the inferior-superior-nasal-temporal (ISNT)-rule of the neuroretinal rim shape was not fulfilled in early glaucoma and in eyes with a normally shaped optic disc (i.e., the rim width at the inferior disc pole or at the superior disc pole was equal to or smaller than the rim width temporal; it included a notch in the neuroretinal rim in the temporal inferior region and / or the temporal superior region); or (2) if an abnormally large cup was present in a small optic disc which normally would not show cupping. In all eyes with glaucoma, the visibility of the retinal nerve fiber layer was locally and / or segmentally reduced. The assessment of the optic disc photographs was carried in a masked manner without knowledge of intraocular pressure or the perimetric results. Each photograph of a glaucomatous optic disc was independently adjudicated by two senior graders (VN and JBJ). The whole glaucoma group was differentiated into subjects with open-angle glaucoma and with primary angle closure glaucoma. Open-angle glaucoma was characterized by an open anterior chamber angle, in addition to a normal depth of the anterior chamber as assessed by slit lamp biomicroscopy. In angle-closure glaucoma, the anterior chamber angle was occluded or occludable. The anterior chamber angle was defined as occludable, if ≥270° of the posterior trabecular meshwork could not be seen upon gonioscopy [15]. In addition, other features for angle-closure glaucoma were iris whirling and glaukomflecken in the anterior subcapsular lens region, in combination with a narrow anterior chamber angle.

For the calculation of a formula to estimate the CSFP, we used the lumbar CSFP measurements obtained in a previous study on 72 patients (mean age: 42.0±13.4 years) who consecutively underwent lumbar puncture for diagnosis and treatment of neurological diseases [12]. The indications for lumbar puncture were peripheral neuropathy, intracranial hypertension, spontaneous intracranial hypotension, cavernous sinus syndrome, meningitis, multiple sclerosis, unilateral ischemic optic neuropathy, unilateral optic neuritis, optic nerve atrophy, and head injury. The mean CSFP was 12.6±4.8 mm Hg. All measured CSFP was less than 25 mmHg in all patients. Out of the total group, we randomly formed a training group consisting of 32 patients, and a group including the remaining 42 patients. Performing a multivariate analysis in the training group with the lumbar CSFP measurements as dependent variable and age, body mass index and blood pressure as independent variables revealed, that CSFP was best described by the formula as CSFP [mmHg] = 0.44×Body Mass Index [kg/m^2^]+0.16×Diastolic Blood Pressure [mmHg]−0.18×Age [Years]−1.91. The association between higher CSFP and younger age, higher body mass index and higher blood pressure had also been found in other previous investigations [16], [17]. We then tested the formula in the independent test group. In this test group, measured lumbar CSFP (12.6±4.8 mm Hg) did not differ significantly (*P* = 0.29) from the calculated CSFP (13.3±3.2 mm Hg). The Durbin-Watson value was 2.08. Values falling into the acceptable range of 1.5 to 2.5 indicate a non-significant autocorrelation for the residuals in the multiple regression models [18]. The intra-class correlation coefficient was 0.71. The Bland-Altman analysis revealed that 40 out of 42 measurements were within the 95% limits of agreement. If the test group was taken as training group, the algorithm to calculate the CSFP was CSFP [mmHg] = 0.85×Body Mass Index [kg/m^2^]+0.27×Diastolic Blood Pressure [mmHg]−0.08×Age [Years]−24.8.

Only those subjects with assessable optic disc photographs and measurements of blood pressure, body height and weight, and IOP were included into the study. Statistical analysis was performed using a commercially available statistical software package (SPSS for Windows, version 21.0, SPSS, Chicago, IL). Continuous data were presented as mean ± standard deviation. In a first step, we examined the mean values. In a second step, we searched for associations between TLCPD, CSFP and other systemic and ocular parameters, first in univariate analysis, and then in multivariate analysis. In a third step, we compared TLCPD and CSFP between glaucomatous subjects and non-glaucomatous subjects and searched for associations between TLCP and CSFP on one side and parameters indicating the amount of glaucomatous optic nerve damage on the other side. In a fourth step, logistic regression analyses were performed to investigate associations of the presence of glaucoma and TLCPD. Odds ratios (OR) and 95% confidence intervals (CI) were presented. All *P*-values were 2-sided and were considered statistically significant when the values were less than 0.05.

## Results

Out of the 9422 eyes (4711 subjects), optic disc photographs and values for TLCPD were available for 8815 (93.6%) eyes of 4546 (96.5%) subjects. The other 165 participants were excluded since assessable photographs of the optic nerve head or values of blood pressure, body mass index or IOP were not available. The mean age of the 4546 participants (53.7% women) was 48.5±12.9 years (median: 45.0 years; range: 30 – 100 years), mean refractive error was −0.13±1.77 diopters (median: 0.00 diopters; range: −21.75 to+6.87 diopters), and mean axial length was 22.64±0.88 mm (median: 22.61 mm; range: 18.22 to 32.70 mm). The group of excluded subjects as compared with the group of subjects included into this study was significantly older (64.9±14.0 years versus 48.5±12.9 years; *P*<0.001) and more myopic (−1.17±2.34 diopters versus −0.13±1.77 diopters; *P*<0.001), while both groups did not differ significantly in axial length (22.81±1.33 mm versus 22.64±0.88 mm; *P* = 0.13), IOP (14.5±6.0 mmHg versus 14.0±3.4 mm Hg; *P* = 0.34) and gender (women: 53.7% versus 46.7%; *P* = 0.08).

Using the optic nerve head criteria for the definition for glaucomatous optic neuropathy, glaucoma was detected in 193 eyes (2.19% (95%CI: 1.88, 2.49) of 122 subjects (51 unilateral, 71 bilateral) (2.7%). According to the gonioscopic finding, the whole glaucoma group was differentiated into an OAG group (n = 173 eyes) and subjects with ACG (n = 20 eyes). Using the criteria of the International Society of Geographical and Epidemiological Ophthalmology, glaucoma was detected in 129 eyes of 121 subjects, with 121 eyes fulfilling the criteria of open-angle glaucoma.

**Figure 1:**
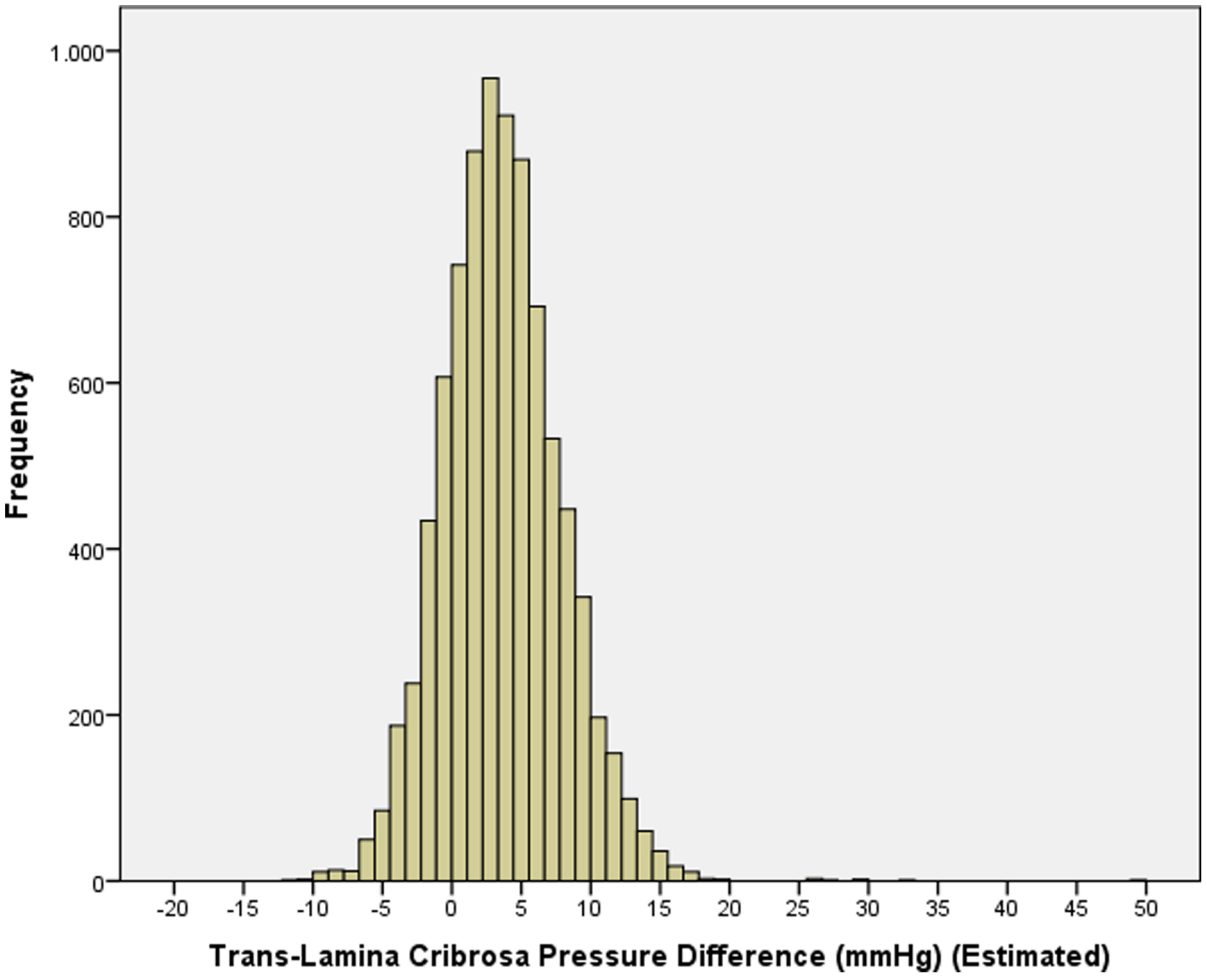
Histogram showing the distribution of the estimated trans-lamina cribrosa pressure difference in the non-glaucomatous eyes of the Central India Eye and Medical Study.

In the non-glaucomatous population, mean TLCPD was 3.64±4.25 mm Hg (median: 3.42 mm Hg) (Fig. 1). In univariate analysis, TLCPD was significantly associated with the systemic parameters of older age (*P*<0.001; correlation coefficient r: 0.52), lower body height (*P*<0.001; r: −0.11), lower body weight (*P*<0.001; r: −0.40), lower body mass index (*P*<0.001; r: −0.42), lower level of education (*P*<0.001; r: −0.25), lower diastolic blood pressure (*P*<0.001; r: −0.37), lower systolic blood pressure (*P*<0.001; r: −0.07), lower pulse (*P*<0.001; r: −0.05), and higher blood concentration of cholesterol (*P* = 0.01; r: 0.03) and lower hemoglobin concentration (*P*<0.001; r: −0.14); and with the ocular parameters of shorter axial length (*P*<0.001; r: −0.05), myopic refractive error (*P*<0.001; r: −0.10), shallower anterior chamber (*P* = 0.001; r: −0.10), thicker lens (*P*<0.001; r: 0.07), thicker central cornea (*P*<0.001; r: 0.05), and higher corneal refractive power (*P*<0.001; r: 0.12). It was not significantly associated with blood concentration of glucose (*P* = 0.13), high-density lipoproteins (*P* = 0.17), and of glycosylated hemoglobin (*P* = 0.91).

The multivariate analysis within the non-glaucomatous group included TLCPD as dependent variable and all those parameters which were significantly associated with TLCPD in the univariate analysis. In a first step, we removed all parameters for which the variance inflation factor as part of the analysis of collinearity was higher than 2.0. We then dropped out of the list of independent parameters those variables, which were no longer significantly associated with TLCPD, starting with the parameters with the highest *P*-values. It resulted in a final model in which TLCPD was associated with the systemic parameters of older age (*P*<0.001), lower body mass index (*P*<0.001), lower diastolic blood pressure (*P*<0.001), higher pulse (*P*<0.001), lower body height (*P*<0.001), higher level of education (*P*<0.001), higher blood concentrations of cholesterol (*P*<0.001), and with the ocular parameters of longer axial length (*P* = 0.001), thicker central cornea (*P*<0.001), and higher corneal refractive power *P*<0.001). If we then added the presence of glaucoma to the list of independent variables, TLCPD was additionally associated with glaucomatous optic neuropathy (Table 1). In a similar manner, smaller neuroretinal rim area was significantly associated with higher TLCPD (*P*<0.001; standardized coefficient beta: −0.06; regression coefficient B = −0.79; 95%CI: −0.99, −0.58) in this multivariate analysis model.

**Table 1:** Results of the multivariate analysis of the association between the calculated trans-lamina cribrosa pressure difference and ocular and systemic parameters in the non-glaucomatous population of the Central India Eye and Medical Study.

Parameter	*P*-Value	Standardized Coefficient Beta	Regression Coefficient B	95% Confidence Interval	Variance Inflation Factor
Age (Years)	<0.001	0.51	0.18	0.17, 0.18	1.29
Body Mass Index (kg/m^2^)	<0.001	−0.28	−0.36	−0.38, −0.33	1.21
Diastolic Blood Pressure (mmHg)	<0.001	−0.31	−0.12	−0.13, −0.11	1.25
Pulse	<0.001	0.05	0.02	0.01, 0.02	1.15
Body height (cm)	0.02	−0.02	−0.01	−0.02, 0.00	1.35
Level of Education	<0.001	0.04	0.15	0.09, 0.22	1.40
Blood Concentration of Cholesterol (mg/dL)	<0.001	0.05	0.01	0.01, 0.01	1.05
Axial Length (mm)	0.006	0.03	0.14	0.04, 0.24	1.65
Central Corneal Thickness (µm)	<0.001	0.15	0.02	0.02, 0.02	1.05
Corneal Refractive Power (Diopters)	<0.001	0.07	0.18	0.13, 0.23	1.60
Prevalence of Glaucoma	<0.001	0.11	3.43	2.96, 3.99	1.04

If the parameters of body mass index, age and diastolic blood pressure were dropped from the multivariate model, since these parameters were included in the formula to calculate the CSFP, TLCPD was significantly associated with a higher prevalence of glaucoma (*P*<0.001; beta: 0.17; B: 5.22; 95%CI: 4.60, 5.84) after adjusting for lower pulse (*P*<0.001; beta: −0.08; B: −0.03; 95%CI: −0.03, −0.02), shorter body height (*P* = 0.001; beta: −0.04; B: −0.02; 95%CI: −0.03, −0.01), lower educational level (*P*<0.001; beta: −0.23; B: −0.80; 95%CI: −0.88, −0.72), longer axial length (*P* = 0.001; beta: 0.04; B: 0.23; 95%CI: 0.10, 0.36), thicker central cornea (*P* = 0.01; beta: 0.10; B: 0.01; 95%CI: 0.01, 0.02), higher corneal refractive power (*P*<0.001; beta: 0.11; B: 0.29; 95%CI: 0.22, 0.36), and higher blood concentration of cholesterol (*P* = 0.05; beta: 0.02; B: 0.003; 95%CI: 0.000, 0.006).

**Figure 2:**
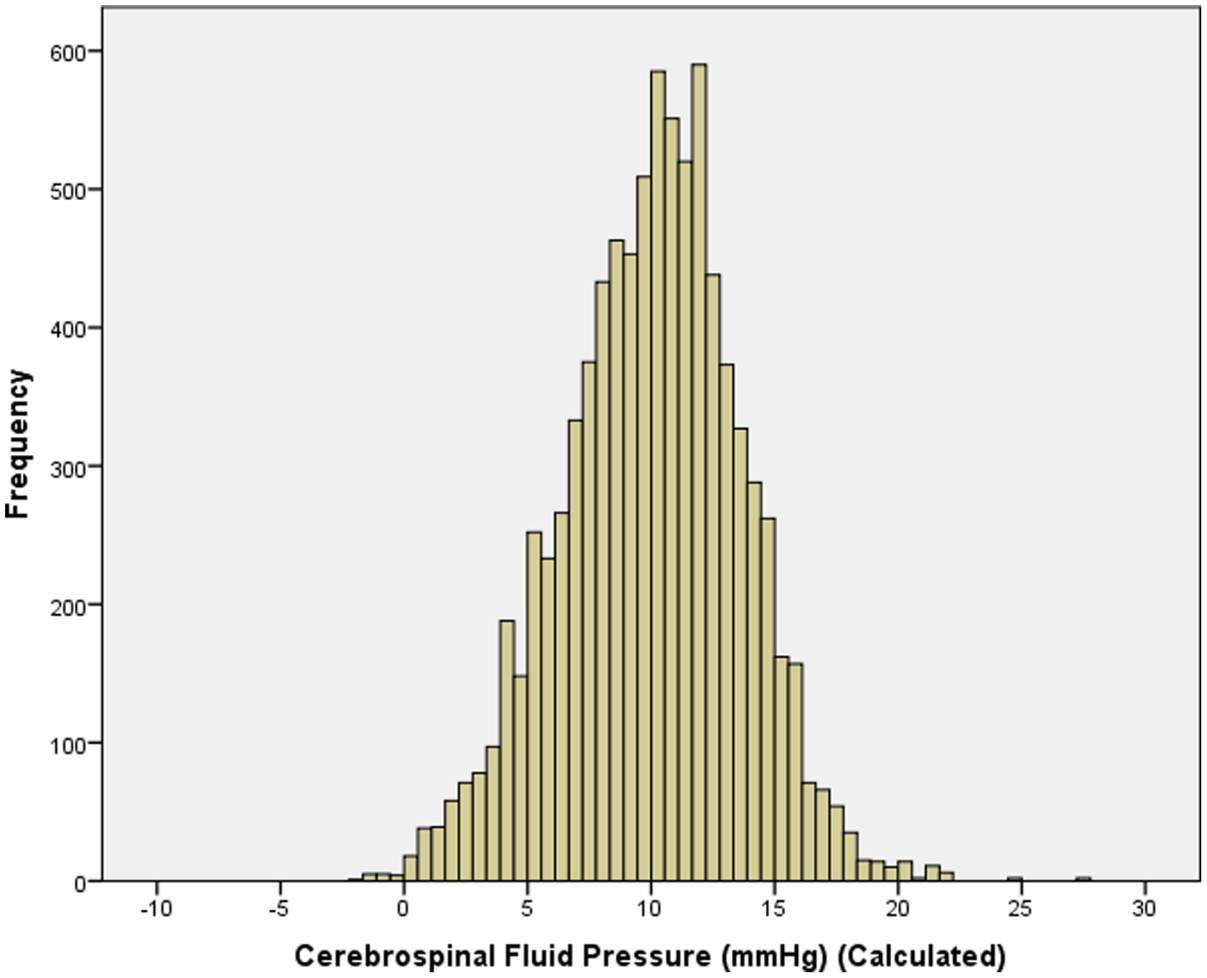
Histogram showing the distribution of the estimated cerebrospinal fluid pressure in the non-glaucomatous eyes of the Central India Eye and Medical Study.

**Figure 3:**
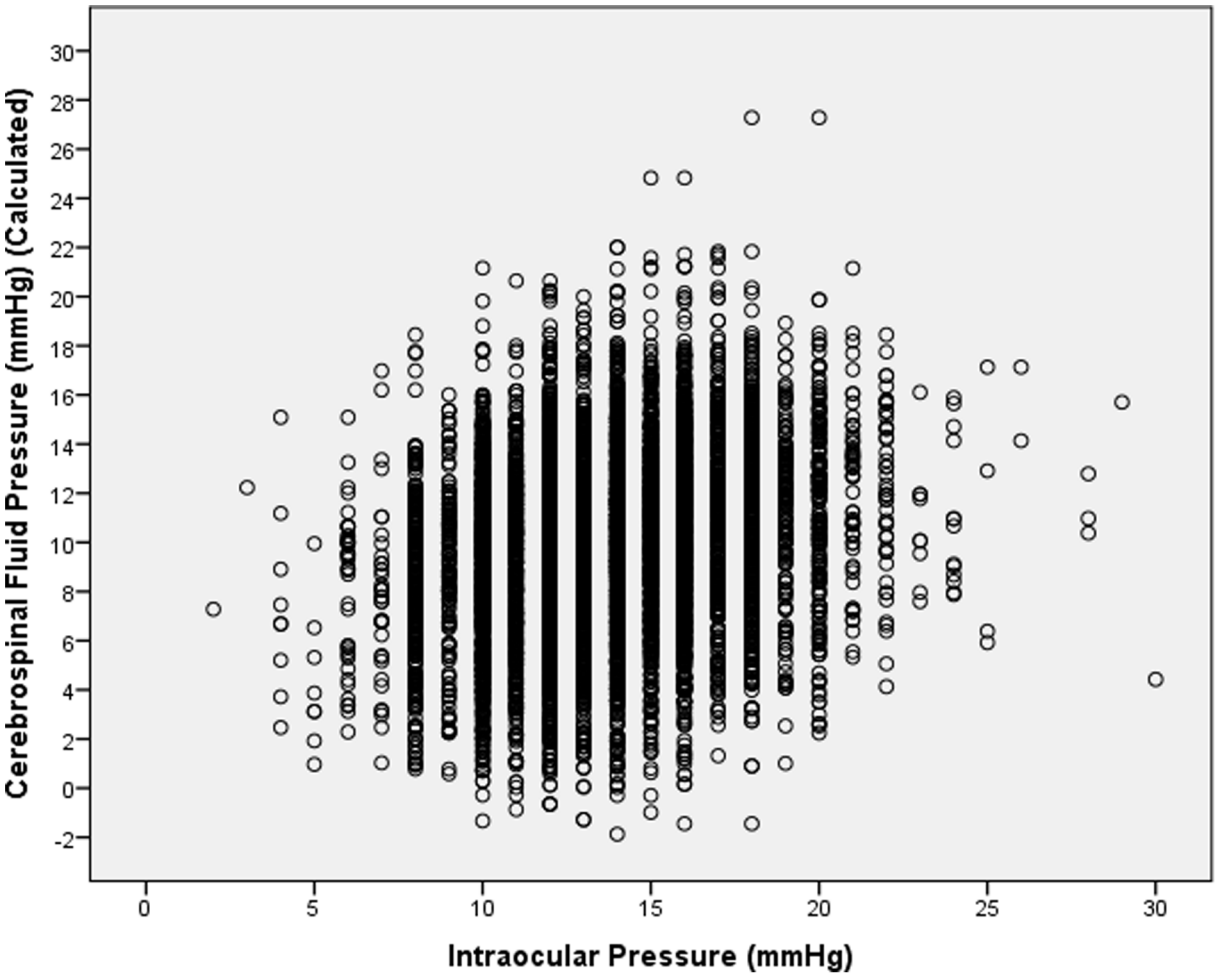
Scattergram showing the correlation between measured intraocular pressure and calculated cerebrospinal fluid pressure in the non-glaucomatous and non-highly myopic population of the Central India Eye and Medical Study. (P<0.001; correlation coefficient r: 0.22; equation of the regression line: Calculated Cerebrospinal Fluid Pressure [mmHg] = 0.25×Intraocular Pressure [mmHg]+6.64 mmHg).

In the non-glaucomatous population, mean estimated CSFP was 10.0±3.6 mm Hg (median: 10.2 mm Hg) (Fig. 2). In univariate analysis, CSFP was significantly associated with younger age (*P*<0.001; r: −0.64), female gender (*P* = 0.049), taller body height (*P*<0.001; r: 0.12), higher body weight (*P*<0.001; r: 0.58), higher body mass index (*P*<0.001; r: 0.64), higher diastolic blood pressure (*P*<0.001; r: 0.62) and systolic blood pressure (*P*<0.001; r: 0.26), higher pulse (*P*<0.001; r: 0.18), higher level of education (*P*<0.001; r: 0.37), and higher blood concentration of cholesterol (*P<*0.001; r: 0.05) and glucose (*P<*0.001; r: 0.09), and with the ocular parameters of higher intraocular pressure (*P*<0.001; r: 0.22) (Fig. 3), thicker central cornea (*P*<0.001; r: 0.13), lower corneal refractive power (*P*<0.001; r: −0.12), refractive error (*P*<0.001; r: 0.07), longer axial length (*P*<0.001; r: 0.07), deeper anterior chamber (*P*<0.001; r: 0.12), and thinner lens (*P*<0.001; r: −0.06). It was not significantly associated with blood concentration of glycosylated hemoglobin (*P* = 0.20) and high-density lipoproteins (*P* = 0.65).

The multivariate analysis included CSFP as dependent variable and all those parameters which were significantly associated with CSFP in the univariate analysis. In a first step, we removed all parameters for which the variance inflation factor as part of the analysis of collinearity was higher than 2.0. We then dropped out of the list of independent parameters those variables, which were no longer significantly associated with CSFP, starting with the parameters with the highest *P*-values. We eventually arrived at a model in which CSFP was significantly associated with younger age (*P*<0.001), higher body mass index (*P*<0.001), higher systolic blood pressure (*P*<0.001), higher pulse (*P*<0.001), taller body height (*P*<0.001), and higher IOP (*P* = 0.03) (Table 2).

**Table 2:** Results of the multivariate analysis of the association between the calculated cerebrospinal fluid pressure and systemic and ocular parameters in the non-glaucomatous population of the Central India Eye and Medical Study.

Parameter	*P*-Value	Standardized Coefficient Beta	Regression Coefficient B	95% Confidence Interval	Variance Inflation Factor
Age (Years)	<0.001	−0.72	−0.20	−0.21, −0.20	1.19
Body Mass Index (kg/m^2^)	<0.001	0.51	0.53	0.52, 0.53	1.09
Systolic Blood Pressure (mmHg)	<0.001	0.38	0.07	0.07, 0.07	1.25
Pulse	<0.001	0.10	0.03	0.03, 0.03	1.08
Body Height (cm)	<0.001	0.03	0.01	0.01, 0.02	1.07
Intraocular Pressure (mmHg)	0.04	0.01	0.01	0.00, 0.02	1.09

If the parameters of body mass index, age and diastolic blood pressure were dropped from the multivariate model (since these parameters were included in the formula to calculate the CSFP), higher CSFP was significantly associated with a lower prevalence of glaucoma (*P*<0.001; beta: −0.08; B: −2.11; 95%CI: −2.57, −1.64) after adjusting for higher pulse (*P*<0.001; beta: 0.14; B: 0.04; 95%CI: 0.03, 0.05), longer body height (*P*<0.001; beta: 0.21; B: 0.08; 95%CI: 0.07, 0.09), female gender (*P*<0.001; beta: 0.27; B: 1.93; 95%CI: 1.74, 2.13), higher educational level (*P*<0.001; beta: 0.35; B: 1.01; 95%CI: 0.96, 1.07), higher blood concentration of cholesterol (*P*<0.001; beta: 0.04; B: 0.005; 95%CI: 0.003, 0.008) and glucose (*P*<0.001; beta: 0.06; B: 0.007; 95%CI: 0.005, 0.010), thicker central cornea (*P*<0.001; beta: 0.04; B: 0.005; 95%CI: 0.003, 0.007), less corneal refractive power (*P*<0.001; beta: −0.05; B: −0.11; 95%CI: −0.16, −0.07), and higher intraocular pressure (*P*<0.001; beta: 0.14; B: 0.15; 95%CI: 0.13, 0.17). If the total glaucoma group was differentiated into OAG and ACG, the absolute value of the standardized coefficient beta in the model was higher for OAG (beta: −0.08) than for ACG (beta: −0.03). If the OAG group was further subdivided into a subgroup with an IOP measurement ≤21 mmHg (“normal-IOP OAG group”; n = 148) and a subgroup with an IOP>21 mmHg (“high-IOP OAG group”; n = 25), the association between higher CSFP and lower glaucoma prevalence in the model described above was better for the normal-IOP OAG group (*P*<0.001; beta: −0.06) than for the high-IOP OAG group (*P* = 0.02; beta: −0.02).

Comparing the whole glaucoma group with the non-glaucomatous group showed that IOP was significantly higher (17.2±7.4 mmHg versus 13.7±3.2 mmHg; *P*<0.001), the estimated CSFP was lower (7.6±3.8 mmHg versus 10.0±3.6 mmHg; P<0.001) and the mean TLCPD was significantly (*P*<0.001) higher (9.8±8.2 mmHg versus 3.6±4.2 mmHg in the glaucoma group. The differences between both groups were highest for TLCPD (6.2 mmHg) followed by IOP (3.5 mmHg) and the estimated CSFP (2.4 mmHg). If the whole glaucoma group was divided into OAG and ACG, the difference between the glaucomatous group and the non-glaucomatous group in IOP was higher for ACG than for OAG (8.5 mmHg versus 3.0 mmHg), while the difference in CSFP between the glaucomatous group and the non-glaucomatous group was more pronounced for the OAG group than for the ACG group (3.0 mm Hg versus 1.8 mmHg). Correspondingly, the correlation coefficients between the pressure parameters and the presence of glaucomatous optic neuropathy were higher for TLCPD than for IOP in the OAG group (r = 0.17 versus r = 0.12), while the correlation coefficients were higher for IOP than for TLCPS in the ACG group (r = 0.11 versus r = 0.12). As a corollary, the association between presence of glaucoma and lower CSFP in binary regression analysis was stronger for OAG (*P*<0.001; B: −0.19; OR: 0.83; 95%CI: 0.79, 0.86) than for ACG (*P* = 0.03; B: −0.13; OR: 0.88; 95%CI: 0.78, 0.99).

A previous study had revealed that the prevalence of OAG was significantly associated with higher age (*P*<0.001), lower body mass index (*P* = 0.008), higher mean blood pressure (*P* = 0.02), prevalence of myopic retinopathy (*P*<0.001), lower level of education (*P* = 0.006), longer axial length (*P* = 0.002), and higher intraocular pressure (*P*<0.001; B =  0.16; OR: 1.17; 95%CI: 1.13, 1.22) [19]. If IOP was replaced by TLCPD, a similar association for TLCPD as for IOP was obtained (*P*<0.001; B =  0.16; OR: 1.17; 95%CI: 1.12, 1.22). If only IOP and TLCPD were included into the analysis, the presence of OAG was significantly associated with TLCPD (*P*<0.001; OR: 1.24; 95%CI: 1.19, 1.29) but not with IOP (*P* = 0.08; OR: 0.96; 95%CI: 0.91, 1.00).

The prevalence of ACG was significantly correlated with older age (*P* = 0.02), higher IOP (*P*<0.001), more hyperopic refractive error (*P*<0.001), shallower anterior chamber (*P* = 0.01), and shorter axial length (*P*<0.001). If TLCPD was added to the model, it was not significantly associated with the presence of ACG (*P* = 0.55). If only IOP and TLCPD were included into the analysis with age as additional independent parameter, the presence of ACG was significantly associated with IOP (*P = *0.04; OR: 1.19; 95%CI: 1.01, 1.40) but not with TLCPD (*P* = 0.92).

If the amount of glaucomatous damage was quantified by neuroretinal area, the association between rim area and smaller TLCPD (*P*<0.001; beta: −0.07; B: −0.01; 95%CI: −0.01, −0.00) was stronger than the association between rim area and IOP (*P* = 0.01; beta: −0.03; B: 0.00; 95%CI: −0.01, 0.00) after adjusting for gender (*P* = 0.03), optic disc area (*P*<0.001) and axial length (*P* <0.001). This difference was more pronounced for the OAG than for the ACG group.

## Discussion

Recent experimental, clinical and anatomical investigations have suggested that the orbital CSFP may be of importance for the physiology and pathophysiology of the optic nerve head and may play a role in the pathogenesis of glaucomatous optic neuropathy [1]–[11]. Anatomical investigations have shown that the orbital CSFP is the trans-laminar cribrosa counter-pressure against the IOP, since the cerebrospinal fluid space extends from the intracranial compartment along the optic nerve as fascicle of the brain into the orbit and ends at the peripapillary scleral flange of the optic nerve head [20]. Consequently, considerations on the physiology of the optic nerve head have suggested that the TLCPD as compared with the trans-corneal pressure difference may be more important for any pressure related pathology of the optic nerve head region. The trans-corneal pressure difference has usually been termed “intraocular pressure”. Retrospective chart analyses and a small-scaled prospective clinical study have suggested that some patients with glaucomatous optic nerve damage and normal intraocular pressure may have an abnormally low CSFP [7]–[10]. The results and conclusions of these studies were supported by observations made in a recent investigation in which the width of the orbital CSF space was significantly smaller in patients with normal-pressure glaucoma than in patients with high-pressure glaucoma or in normal subjects [11]. The orbital CSF space width depends on the intracranial CSFP [12].

Using a formula to estimate CSFP and TLCPD, our population-based study revealed that TLCP as compared with IOP was better associated with the presence and amount of glaucomatous optic nerve damage in patients with OAG. In patients with ACG, however, IOP as compared with TLCPD was better correlated with the presence of glaucoma. These results agreed with previous investigations in which an abnormally low (orbital) CSFP was associated with the presence and severity of OAG [7]–[11]. Since ACG is characterized by a markedly elevated IOP due to intraocular reasons, the finding that presence and amount of glaucomatous damage in patients with ACG was better correlated with IOP than TLCPD agreed with the hypothesis that in OAG extraocular factors such as CSFP may be involved in the pathogenesis of the disease. In that context, it may have to be taken into account that ACG may not solely be IOP dependent, as there are patients with ACG and very good IOP control undergoing visual field deterioration.

Interestingly, using the formula described above resulted in a mean value of CSFP of 10.0±3.6 mm Hg (median: 10.2 mm Hg) which is similar to the reported mean CSFP as measured by lumbar puncture [7]–[11], [21]. If one uses the mean and two-fold standard deviation of this value to calculate the range in which 95% of the measurements may be with expected (assuming a Gaussian distribution), this range would reach from 2.8 mm to 17.2 mm Hg. It may be of interest that the maximum value of this range is about 6 times larger than the minimal value, while in the case of IOP (range: 10–21 mmHg), the maximum value is only double as high as the minimal range. The considerably larger relative range of estimated CSFP versus IOP may point to the possibility that subjects with an elevated TLCPD may be more likely to have a relatively low CSFP than to have a relatively high IOP. This finding may be of interest for the discussion of a low CSFP as reason for glaucomatous optic neuropath yin patients with normal-(intraocular)pressure glaucoma.

In the non-glaucomatous group, CSFP was significantly (*P*<0.001) correlated with IOP (Fig. 3). It agrees with a recent study in which CSFP as measured by lumbar puncture was significantly associated with IOP [9]. For both studies, the equations of the regression lines (CSFP [mmHg] = 0.57×IOP [mmHg]+4.7 mmHg) versus (CSFP [mmHg] = 0.25×IOP [mmHg]+6.64 mmHg) did not vary profoundly.

If, in a parallel manner to CSFP, one calculates for the TLCPD (mean: 3.64±4.25 mm Hg) the range in which 95% of the measurements are to be expected (assuming a Gaussian distribution), the range will go from −4.9 mmHg to 12.1 mmHg. This range in relative terms is even larger than the normal range for CSFP. If one takes into account that TLCPD is more important than CSFP for the pressure conditions in the optic nerve head, the large normal range of TLCPD may exemplify the role alterations of the TLCPD may potentially play in the pathophysiology of the optic nerve head.

In multivariate analysis in the non-glaucomatous study population, higher TLCPD was associated with among other parameters, with older age, lower body mass index, lower diastolic blood pressure, and more myopic refractive error (Table 1). These results fit well with the findings obtained in previous hospital-based investigations and population-based studies in which the prevalence of open-angle glaucoma was associated with older age, myopia, lower blood pressure and lower body mass index [19], [22], [23].

Potential limitations of our study should be mentioned. First, the whole statistical analysis depended on the formula to calculate the CSFP. The study, in which the basis parameter for that formula were assessed included a relatively small number of subjects, and these subjects had a clinical reason to undergo lumbar puncture [12]. Although the clinical neurological examination and the further clinical course retrospectively revealed that it was unlikely that the lumbar CSFP measurement in that study group was markedly influenced by the reason to perform the lumbar puncture, one has to keep in mind, that the participants in that study were not randomly selected normal subjects. In view of this weakness in the study design, one may however, also take into account, that it would not have been possible to measure the CSFP in a population-based study. Second, as for any population-based study, the rate of non-participation or non-availability of examination results can matter. In our study, the participation rate was 80.1% what may be acceptable. Third, IOP was measured only once, so that the question arises how representative this single IOP measurement was for the subject's IOP in general. Consequently, it was only an attempt to differentiate within the OAG group between patients with high IOP and subjects with normal IOP. Fourth, the formula to estimate the CSFP was derived in a study on Chinese, and it has remained unclear whether this formula based on Chinese subjects can be transferred to Indians. Up to now, however, ethnic differences in the normal CSFP have not been reported yet. Fifth, the statistical analysis for the ACG group was limited by the relatively small sample size in that subgroup (n = 20). Fifth, the design of our investigation as a cross-sectional study does not rule possibilities of a dynamic changes in CSFP and TLCPD with age so that the causativeness of TLCPD with glaucoma subtypes has to be further examined in longitudinal studies.

In conclusion, in OAG but not in ACG, calculated TLCPD versus IOP showed a better association with presence of glaucoma and amount of glaucomatous optic neuropathy. It supports the notion of a potential role of low CSFP in the pathogenesis of OAG. In contrast, IOP as compared with TLCPD was better correlated with presence and amount of glaucomatous damage in ACG, fitting with the notion that the intraocular reason for the elevation of IOP, and not the retro-laminar pressure, was the major driving cause for glaucomatous optic neuropathy in ACG.
